# Telemedicine in Pediatric Clinics: Parental Satisfaction and Perception at King Khalid University Hospital During the COVID-19 Pandemic

**DOI:** 10.7759/cureus.78721

**Published:** 2025-02-07

**Authors:** Ahmed I Albarrak, Muan M Almoajil, Meshal A Alqahtani, Nawaf A Alshehri, Naif H Alhasan, Abdullah S Al-Ibrahim, Abdulaziz M Alothman

**Affiliations:** 1 College of Medicine, King Saud University, Riyadh, SAU

**Keywords:** covid-19, covid-19 telemedicine, parents, perception, satisfaction, telemedicine

## Abstract

Background

Telemedicine enables healthcare professionals to provide clinical services through digital applications and communication technologies remotely. Previous studies have reported a significant increase in telemedicine usage during the COVID-19 pandemic, particularly in Saudi Arabia. As the global adoption of telemedicine services continues to rise, patient satisfaction remains a key quality indicator. This study aimed to evaluate parental satisfaction with telemedicine services in pediatric clinics at King Khalid University Hospital (KKUH) during the COVID-19 pandemic. Additionally, it sought to assess parents’ perceptions of telemedicine in this setting.

Methods

This descriptive cross-sectional study included 412 parents who used telemedicine services during the COVID-19 pandemic in pediatric clinics at KKUH in Riyadh, Saudi Arabia. A validated online questionnaire, adapted from a previous study, was used for data collection. The questionnaire comprised four main sections: informed consent, sociodemographic questions, satisfaction statements, and perception statements.

Results

The study included 412 participants, with a response rate of 56%. Among the satisfaction domains, the highest rated was safety (86%), followed by appropriateness (84%), access and timeliness (84%), effectiveness (82%), and efficiency (77%). Additionally, a positive correlation was observed between the level of satisfaction across these domains and parental perception (p < 0.05).

Conclusions

The study concluded that although parents were highly satisfied with the use of telemedicine during the COVID-19 pandemic, their overall perception of telemedicine was less favorable.

## Introduction

Telemedicine is defined as “the utilization of computer applications and telecommunication technologies to deliver clinical services remotely” [1]. It offers numerous advantages over traditional in-person healthcare for both patients and providers. These benefits include reduced travel costs and time, minimized waiting room durations, a lower risk of infectious disease transmission, improved convenience, and more efficient care delivery [2].

During the COVID-19 pandemic, there was a significant surge in the adoption of telemedicine and other healthcare interventions [3]. In the United States, approximately two-thirds of healthcare practitioners utilized telemedicine during the pandemic, with one-fifth expressing an intention to continue its use afterward [3]. Similarly, most pediatric surgeons in the United States incorporated telemedicine into their routine clinical practices [4].

A recent study in Saudi Arabia found that 93.1% of respondents agreed that telehealth services improved healthcare accessibility and expressed willingness to participate in future telemedicine consultations [5]. In the United Kingdom, another study reported high satisfaction with telemedicine among both patients and healthcare providers [6].

In Saudi Arabia, two-thirds of physicians utilized telemedicine during the pandemic, with nine out of 10 using it for follow-ups with chronic disease patients at home [7]. The most commonly perceived advantages of telemedicine among Saudi patients included time savings, provider availability, and convenience [8]. A survey of randomly selected Saudi citizens revealed that two-thirds were familiar with the concept of telemedicine, and one-third had used it before. During the pandemic, the vast majority recognized its benefits [9].

Patients’ perception plays a crucial role in determining the future adoption of telemedicine. Understanding patient perceptions, including personal, social, and cultural factors, can enhance its usability and encourage broader acceptance [8,10]. However, patient satisfaction is equally vital, as it serves as a key quality indicator in assessing the effectiveness of healthcare services, including telemedicine. Dissatisfaction can lead to reduced efficiency and increased healthcare costs [8]. Given the global rise in telemedicine utilization during the COVID-19 pandemic, monitoring patient satisfaction remains essential [8].

In Saudi Arabia, 70% of respondents acknowledged the benefits of telemedicine [10]. Previous studies have highlighted the increased adoption of telemedicine during the pandemic, particularly in Saudi Arabia [8,11], and a growing willingness among patients to continue using it post-pandemic [8]. However, based on our literature review, there is a lack of local research measuring parental satisfaction and perception of telemedicine in pediatric clinics. Prior studies have recommended a comprehensive evaluation of telemedicine satisfaction and perception, particularly in specific populations such as children [10].

This study aims to assess the satisfaction and perception of parents regarding the use of telemedicine in pediatric clinics at King Khalid University Hospital (KKUH).

## Materials and methods

This descriptive cross-sectional study included 412 parents who utilized telemedicine services during the COVID-19 pandemic in pediatric clinics at KKUH in Riyadh, Saudi Arabia. Participants were parents of pediatric patients who attended virtual outpatient appointments via video or phone calls between March 2020 and September 2020. Parents who declined to participate were excluded from the study.

Ethical approval was obtained from the Institutional Review Board of the College of Medicine, King Saud University (KSU) (approval number E-22-7051, issued on August 22, 2022). Data collection took place between September and October 2022.

This study employed a descriptive cross-sectional design, collecting data at a single point in time to assess parental satisfaction and perception based on retrospective experiences, without tracking participants longitudinally. A simple random sampling technique was used after obtaining permission from the pediatric department to access the medical record numbers and phone numbers of patients who attended telemedicine clinics during the study period.

Sample size calculation

The required sample size was estimated using the following formula:

\[n = \frac{Z^2 p (1 - p)}{d^2},\]

where Z = 1.96 at a 95% CI, p = 0.68 (prevalence of satisfaction with telemedicine based on a previous study [12]), 1 - p = 0.32, and d = 0.05 (margin of error). Substituting these values, the calculated sample size was 334. To account for a 20% non-response rate, the final target sample size was set at 401. Using a simple random sampling technique, we successfully recruited 412 participants.

Data collection

A validated online questionnaire was distributed to 736 parents via WhatsApp (Meta Platforms, Inc., Menlo Park, California, USA) using the Google Forms platform (Google LLC, Mountain View, California, USA). The questionnaire was adapted from a previous study [13] and translated into Arabic. To ensure linguistic and conceptual accuracy, it was reviewed by experts. A pilot test was conducted with ten participants to assess clarity and comprehensiveness; these participants were excluded from the final sample. The pilot study confirmed that the questionnaire was well understood, requiring no further modifications. A total of 412 responses were received, yielding a response rate of 56%.

Study variables and questionnaire structure

The study examined exposure variables, including age, gender, education level, transportation to hospital appointments, income, distance from home to the hospital, and internet access at home. The primary outcome variables were parental satisfaction and perception of telemedicine services.

The questionnaire consisted of four sections: (1) informed consent; (2) sociodemographic questions; (3) satisfaction statements; and (4) perception statements. Satisfaction statements were assessed using a four-point Likert scale (1 = “Strongly agree” to 4 = “Strongly disagree”). Neutral options were omitted to minimize central tendency bias, encouraging participants to take a clear stance and ensuring a more definitive assessment of satisfaction levels. While this approach may influence response distribution, it does not compromise validity, as all participants were provided with balanced options for agreement or disagreement. The perception statements were measured using a five-point Likert scale (1 = “Always” to 5 = “Never”).

Data analysis

Statistical analysis was conducted using IBM SPSS Statistics for Windows, Version 26.0 (Released 2019; IBM Corp., Armonk, NY, USA). Frequencies and percentages were used to describe categorical variables. Pearson correlation analysis was performed to assess relationships between satisfaction, perception, and sociodemographic variables, with statistical significance set at p < 0.05.

Ethical considerations

Participant responses were fully anonymous, and informed consent was obtained from all parents before participation. The data was used exclusively for research purposes, and no incentives were provided.

## Results

More than half of the study participants were male (53.4%), while 46.6% were female. The average age of respondents was 42 ± 9 years. Among the age groups, the 40-49 age group had the highest representation, accounting for 38.3% of participants, followed by those aged 30-39 years (30.1%). In contrast, the least represented age groups were 60 years or older (3.4%), followed by 20-29 years (8.3%). Regarding educational background, nearly half of the participants held a bachelor’s degree (48.8%), followed by those with a high school education (20.1%).

Table 1 presents the distribution of the socio-economic characteristics of the study participants. The most common income group was 9,000-13,999 SR (31.6%), while the smallest proportion of participants reported earning more than 24,000 SR (7.0%). In terms of travel time to the hospital, the largest proportion of participants resided 21-30 minutes away (26.2%), whereas the smallest proportion lived 51-60 minutes away (4.6%). Nearly all participants (98.8%) reported having internet access at home.

**Table 1:** Distribution of socioeconomic characteristics of study subjects

Characteristics	N	%
Transportation to hospital appointments		
Personal car	273	66.3
Ride from a family member or friend	104	25.2
Uber/taxi	33	8
No transportation	2	0.5
Monthly income (SR)		
1,000-3,999	47	11.4
4,000-8,999	101	24.5
9,000-13,999	130	31.6
14,000-18,999	73	17.7
19,000-23,999	32	7.8
>24,000	29	7
Distance from home to hospital (minutes)		
Less than 10 minutes	22	5.3
10-20 minutes	95	23.1
21-30 minutes	108	26.2
31-40 minutes	75	18.2
41-50 minutes	51	12.4
51-60 minutes	19	4.6
More than 60 minutes	42	10.2
Internet access at home		
Yes	407	98.8
No	5	1.2

**Figure 1:**
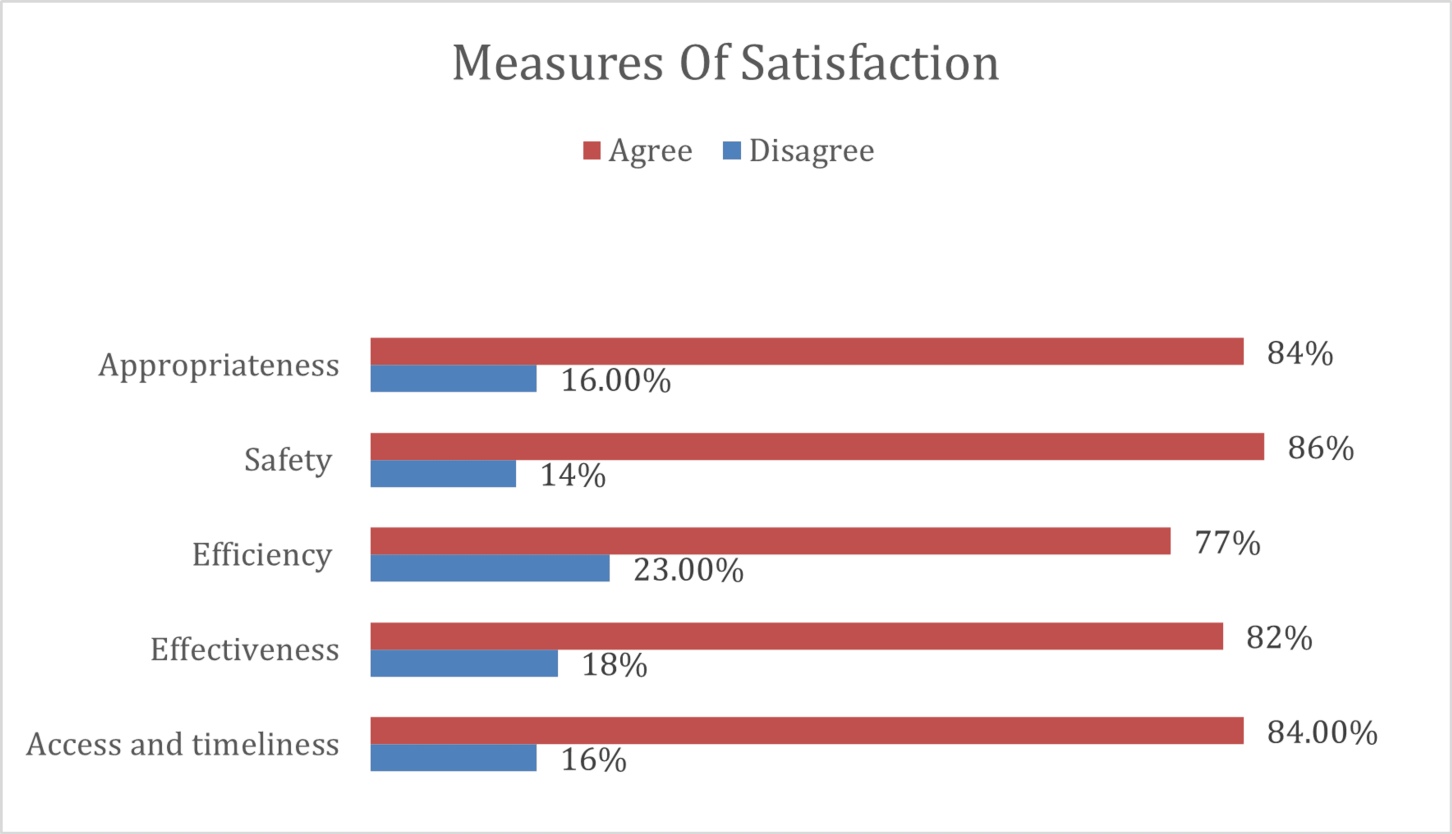
Distribution of satisfaction domains

Table 2 presents the distribution of study participants based on their satisfaction responses. The questions were categorized into five domains, with Figure 1 illustrating the results across these domains. Parental satisfaction was highest in the safety domain (86%), followed by appropriateness (84%), access and timeliness (84%), effectiveness (82%), and efficiency (77%). The findings were statistically significant, with 49.0% of participants strongly agreeing that they felt confident in following the doctor recommendations and 52.9% strongly agreeing that the doctor treated them with courtesy and respect. Additionally, 35.0% strongly agreed and 33.0% agreed that they were able to see the doctor clearly. Moreover, 35.0% strongly agreed and 39.3% agreed that they could access a telemedicine appointment more quickly than an in-person visit.

**Table 2:** Distribution of parents' satisfaction scores on the use of telemedicine in pediatric clinics

Satisfaction statements	Number of survey responses, n	Strongly disagree, n (%)	Disagree, n (%)	Agree, n (%)	Strongly agree, n (%)
Factor 1: access and timeliness	412	18.75 (4.23)	49 (11.88)	183.5 (44.55)	162 (39.3)
Q1. I am satisfied with the length of time I had to wait between my referral and my telemedicine appointment.	412	29 (7.0)	76 (18.4)	171 (41.5)	136 (33.0)
Q2. It was easy to book my telemedicine appointment.	412	16 (3.9)	66 (16.0)	187 (45.4)	143 (34.7)
Q3. The environment of the telemedicine appointments was convenient for me.	412	15 (3.6)	36 (8.7)	194 (47.1)	167 (40.5)
Q4. I am confident that I will be able to follow the doctor recommendation.	412	10 (2.4)	18 (4.4)	182 (44.2)	202 (49.0)
Factor 2: Effectiveness	412	19.67 (4.8)	52 (12.63)	162 (39.3)	178.3 (43.3)
Q5. During my telemedicine appointment, I was able to see the doctor clearly.	412	39 (9.5)	93 (22.6)	136 (33.0)	144 (35.0)
Q6. During my telemedicine appointment, I was able to hear the doctor clearly.	412	11 (2.7)	28 (6.8)	179 (43.4)	194 (47.1)
Q7. I am confident that the doctor and my health care providers are working as a team.	412	9 (2.2)	35 (8.5)	171 (41.5)	197 (47.8)
Factor 3: Efficiency	412	22.5 (5.45)	70.5 (17.1)	165.5 (40.15)	153.5 (37.3)
Q8. I feel that there was an adequate amount of time allotted for my telemedicine appointment.	412	16 (3.9)	64 (15.5)	169 (41.0)	163 (39.6)
Q9. I was able to get an appointment through telemedicine sooner than an in-person appointment.	412	29 (7.0)	77 (18.7)	162 (39.3)	144 (35.0)
Factor 4: Safety	412	16.5 (4.0)	41 (10)	180.75 (43.85)	173.75 (42.2)
Q10. I felt comfortable during my telemedicine appointment.	412	9 (2.2)	42 (10.2)	186 (45.1)	175 (42.5)
Q11. I felt that confidentiality was protected throughout my telemedicine appointment.	412	12 (2.9)	23 (5.6)	186 (45.1)	191 (46.4)
Q12. The doctor explained the benefits and risks of any medications/treatment plan he/she recommended to me.	412	15 (3.6)	43 (10.4)	181 (43.9)	173 (42.0)
Q13. I understood what to do if I have an emergency following this appointment.	412	30 (7.3)	56 (13.6)	170 (41.3)	156 (37.9)
Factor 5: Appropriateness	412	18.3 (4.45)	48.17 (11.7)	169.3 (41.1)	176.17 (42.77)
Q14.I believe telemedicine is just as effective as an in-person appointment.	412	52 (12.6)	112 (27.2)	125 (30.3)	123 (29.9)
Q15. The doctor understood my concerns.	412	7 (1.7)	42 (10.2)	166 (40.3)	197 (47.8)
Q16. The doctor treated me with courtesy and respect.	412	6 (1.5)	16 (3.9)	172 (41.7)	218 (52.9)
Q17. The doctor explained my diagnosis in a way that I could understand.	412	10 (2.4)	27 (6.6)	184 (44.7)	191 (46.4)
Q18. The doctor involved me in decisions about my treatment plan.	412	15 (3.6)	49 (11.9)	183 (44.4)	165 (40.0)
Q19. Overall, I am satisfied with telemedicine service quality.	412	20 (4.9)	43 (10.4)	186 (45.1)	163 (39.6)

Table 3 summarizes the distribution of study participants based on their responses regarding perception. The highest percentages recorded were for statement (2), “I search for information online,” where 51.5% (212 participants) responded “Always,” followed by 28.4% (117 participants) selecting “Often.” In contrast, for statement (4), “I interact with my doctor via email or through social media,” the most common response was “Never” (29.4%, 121 participants), while “Often” had the lowest percentage at 14.6% (60 participants). For statement (5), “I use electronic services provided by the hospital,” the most frequent response was “Sometimes” (23.5%, 97 participants), whereas “Always” had the lowest percentage at 18.2% (75 participants).

**Table 3:** Distribution of parents’ perception scores on the use of telemedicine in pediatric clinics

Perception statements	Number of survey responses, n	Never, n (%)	Rarely, n (%)	Sometimes, n (%)	Often, n (%)	Always, n (%)
Q1. I use a PC/laptop at home.	412	41 (10)	52 (12.6)	88 (21.4)	79 (19.2)	152 (36.9)
Q2. I search for information online.	412	6 (1.5)	13 (3.2)	64 (15.5)	117 (28.4)	212 (51.5)
Q3. I shop online.	412	18 (4.4)	55 (13.3)	106 (25.7)	109 (26.5)	124 (30.1)
Q4. I interact with my doctor via email or through social media.	412	121 (29.4)	92 (22.3)	64 (15.5)	60 (14.6)	75 (18.2)
Q5. I use electronic services provided by the hospital.	412	78 (18.9)	85 (20.6)	97 (23.5)	77 (18.7)	75 (18.2)

Correlation between domains among the study participants

The results indicated a moderate positive correlation between perception and level of satisfaction (r = 0.386), which was statistically significant (p = 0.001). However, no statistically significant correlations were observed among the remaining study variables.

## Discussion

This study aimed to assess parents’ satisfaction and perception of telemedicine use in pediatric clinics at KKUH during the COVID-19 pandemic. Several studies have highlighted that telemedicine became increasingly essential during the pandemic to minimize physical contact with patients [14]. In Saudi Arabia, telemedicine utilization surged during the COVID-19 pandemic [11], with the Ministry of Health launching multiple telehealth, telemedicine, and mobile applications to enhance healthcare accessibility [15].

In this study, nearly all participants (98.8%) had internet access, aligning with a report from the Saudi Communications, Space and Technology Commission, which indicated a 99% internet penetration rate [16]. Additionally, 80% of participants found booking telemedicine appointments easy, a finding consistent with a previous Saudi study where 52% of participants were “very satisfied” and 31.8% were “satisfied” with the registration and scheduling process [11]. Another study in Saudi Arabia reported high satisfaction levels in the domains of service appropriateness (73.7%) and access and timeliness (68.6%) [17]. These findings emphasize that a smooth booking process and accessible services are critical to enhancing overall patient satisfaction with telemedicine.

Most participants expressed confidence in following their physician’s recommendations, supporting the appropriateness of telemedicine [6]. However, only 60% agreed that telemedicine sessions were as effective as in-person consultations. Notably, about 40% of participants had never or rarely used electronic services provided by the hospital, highlighting a significant gap in digital engagement. This suggests that many patients may not be utilizing available digital resources to improve healthcare access and communication. Possible explanations include a lack of awareness about telemedicine services, limited digital literacy, cultural preferences for face-to-face consultations, and concerns regarding privacy and data security. Additionally, some patients may perceive digital interactions as impersonal or insufficient for complex medical concerns.

Several studies in Saudi Arabia have reported high satisfaction levels with telemedicine. For example, a study in a diabetes clinic found that 97% of participants considered telemedicine an essential tool for maintaining glucose control during the pandemic [18]. Similarly, a study in ENT clinics reported that 83% of patients were satisfied with telemedicine services [19]. These findings align with the present study, which found an average satisfaction level of 82.6%.

Internationally, in developed countries such as Australia, approximately 62% of telehealth users reported satisfaction, describing their experience as comparable to or better than in-person clinic visits [20]. In contrast, awareness of telemedicine and its benefits remains limited in many developing countries [21]. Telemedicine is particularly beneficial in rural areas, as it eliminates the need for long-distance travel, saves time and costs, and ensures equitable healthcare access [22]. The high satisfaction levels observed in this study may be attributed to the relatively high proportion (63.6%) of participants with a university education or higher. Higher education levels often correlate with greater technological familiarity and an increased likelihood of adapting to telemedicine platforms. This aligns with previous research indicating that higher education positively influences patient satisfaction with telemedicine [8,12].

This study also revealed that 52% of participants never or rarely interacted with their physicians via email or social media, suggesting a preference for traditional communication methods and a lack of awareness regarding the benefits of digital interactions. Digital communication can enhance healthcare access, convenience, and patient engagement. However, cultural attitudes favoring in-person consultations and concerns about confidentiality may influence preferences. Awareness campaigns could help address these barriers. Similar findings were reported in another Saudi study, where half of the participants had never used their phones to seek medical advice [10]. Conversely, a recent study on head and neck cancer patients in Saudi Arabia found that 99.2% were interested in using mobile tools for communication with healthcare providers, highlighting the growing potential of telemedicine to improve healthcare accessibility [23].

To increase telemedicine adoption, targeted interventions should focus on improving digital literacy through community-based campaigns and patient education programs. Healthcare providers should receive training in effective virtual communication to foster trust in telemedicine services. Policymakers should also address privacy concerns by enhancing data security measures and ensuring transparency in telemedicine platforms. Additionally, user-friendly telemedicine interfaces with multilingual support and culturally appropriate communication strategies could improve engagement and accessibility.

This study is the first to evaluate telemedicine satisfaction and perception among parents in pediatric clinics in Saudi Arabia, offering valuable insights into an underexplored area. The study's strengths include the use of a structured, validated questionnaire, ensuring response reliability, and a relatively large sample size, enhancing its representativeness. Additionally, its focus on real-world telemedicine experiences during COVID-19 provides practical implications for future healthcare strategies.

However, several limitations should be considered. First, data collection relied on self-reported questionnaires, which may introduce response bias due to social desirability or recall issues. Second, the study primarily employed quantitative methods, limiting qualitative insights into dissatisfaction with telemedicine. Additionally, recruitment via WhatsApp may have introduced selection bias, potentially excluding individuals with lower digital literacy or those not using WhatsApp, affecting the generalizability of findings. Furthermore, as this was a single-center study, its results may not be applicable across Saudi Arabia. Future multicenter studies are recommended to validate these findings. Incorporating qualitative research methods, such as in-depth interviews or focus groups, could provide a more comprehensive understanding of patient concerns and preferences.

## Conclusions

This study highlights the high levels of satisfaction among parents using telemedicine in pediatric clinics during the COVID-19 pandemic. However, gaps in perception and engagement, particularly in digital communication between patients and physicians, underscore the need for targeted interventions. Policymakers should implement structured digital literacy programs to equip patients with the skills needed to navigate telemedicine effectively. Additionally, healthcare institutions should strengthen physician-patient communication by developing secure, user-friendly telemedicine platforms. Addressing privacy concerns through robust data protection policies is essential to fostering trust in virtual healthcare services. Future research should focus on integrating telemedicine into standard healthcare frameworks, incentivizing its adoption, and evaluating long-term outcomes to optimize its role in pediatric care.
